# The Etiological Profile of Global Developmental Delay at a Tertiary Care Hospital in India: An Observational Study

**DOI:** 10.7759/cureus.41066

**Published:** 2023-06-28

**Authors:** Amulya R Sharma, Mohd Saeed Siddiqui, Suvarna Magar, Ajay Kale, Madhurasree Nelanuthala, Surya Pratap Singh

**Affiliations:** 1 Department of Paediatrics, Mahatma Gandhi Mission's (MGM) Medical College and Hospital, A Constituent Unit of MGM Institute of Health Sciences, Aurangabad, IND

**Keywords:** developing countries, hypoxic ischemic encephalopathy, genetic abnormalities, gdd, etiological yield, etiological profile, global developmental delay

## Abstract

Background

Global developmental delay (GDD) is common and has a significant impact on affected children, families, and society. Understanding its etiology is crucial for management and prevention strategies. However, data on the etiological profile of GDD in developing countries are limited. This study aimed to identify the etiological profile of GDD at a tertiary care hospital in India.

Methodology

This observational study included children aged three months to five years with a developmental quotient below 70%. Data on demographics, clinical features, relevant investigations, and diagnoses were collected. Etiologies were categorized into prenatal, perinatal, postnatal, and unknown causes. Informed consent was obtained from the parents.

Results

A total of 52 children, with a median age of 15.5 months, were included in the study, with 69.2% being males. Prenatal causes accounted for half of the cases, with genetic abnormalities (32.7%) and chromosomal abnormalities (7.7%) being prominent. Perinatal causes were the next most common (34.6%), including hypoxic-ischemic encephalopathy (26.7%). Postnatal causes were rare (3.8%). The overall etiological yield was 88.4%, with some cases remaining unidentified.

Conclusions

Prenatal causes, including genetic and chromosomal abnormalities, are common in GDD. The utilization of genetic testing enhances etiological yield. Hypoxic-ischemic encephalopathy remains a significant factor and highlights the importance of perinatal care in preventing developmental delays. Large multicentric studies are needed for a comprehensive database of etiological profiles.

## Introduction

The global prevalence of global developmental delay (GDD) in children under five years is estimated to range from 1% to 3% [1]. GDD is characterized by significant delays in multiple developmental domains, including motor skills, speech and language, cognition, social/personal development, and activities of daily living [2]. The Global Burden of Disease Study in 2016 reported that there were 52.9 million children under the age of five years with developmental disabilities worldwide, with higher rates observed in developing countries [3]. The impact of GDD on affected children, their families, and society at large is substantial, leading to long-term impairments in academic performance, social interactions, and overall quality of life.

To effectively manage, intervene, and implement preventive strategies, a thorough understanding of the etiological factors contributing to GDD is essential. The etiology of GDD is multifactorial and can arise from various genetic, environmental, and prenatal factors [1]. Recent advancements in genetic technologies, such as next-generation sequencing, have increased the diagnostic yield for identifying the underlying genetic causes of GDD [4]. However, there is a notable lack of comprehensive studies examining the etiological profile of GDD in developing countries, where the burden is often higher due to limited healthcare access, increased prevalence of risk factors, and socioeconomic disparities. Therefore, the objective of this study is to investigate the etiological profile of GDD at a tertiary care hospital in India.

## Materials and methods

Study design

An observational descriptive study was conducted that aimed to investigate the etiology of global developmental delay at a tertiary care hospital in India, between January 2021 to June 2022.

Study participants

We included children aged between three months and five years who were identified to have GDD with a developmental quotient (DQ) < 70%. Children with a preexisting diagnosis, who had a history of head injury, or children whose delay in two or more domains could be explained primarily by motor delay or severe uncorrected visual or hearing impairment were excluded from the study. A convenience sampling technique was used to include patients in the study.

Data collection

We collected data on the demographic characteristics, clinical history, results of the investigations, and etiology of global developmental delay in a predesigned proforma. Etiologies were categorized into prenatal, perinatal, postnatal, and unknown causes. The DQ was calculated by the same pediatric resident doctor for all children by screening developmental milestones and averaging developmental age across all the domains. Hematological investigations, such as complete blood count, were done on a five-part ADIVA 2120i Coulter Analyzer (Siemens Healthineers, Mumbai, India). Patients presenting with microcephaly, macrocephaly, dysmorphic features, focal neurological signs, or suspected neurological injury or malformation underwent neuroimaging using a Philips Multiva 1.5T magnetic resonance imaging (MRI) system (Philips India Limited, Mumbai). Patients with a history of convulsions underwent electroencephalography by T20 Channel Machine. Urine gas chromatography-mass spectroscopy (GCMS), tandem mass spectroscopy (TMS), and plasma and urine amino acids were tested in suspected cases of inborn errors of metabolism. Chromosomal analysis and exon or whole-genome sequencing were performed to find underlying chromosomal or single gene defects in unexplained GDD with or without dysmorphisms or major congenital anomalies. The AI model, ChatGPT developed by OpenAI, was used for improving the academic phrasing of this paper (Appendix).

Data analysis

The collected data were entered into a spreadsheet. The frequency and percentage of the different etiologies were calculated. Statistical analyses were performed using IBM SPSS Statistics for Windows, Version 24.0 (IBM Corp., Armonk, NY, USA).

Ethical considerations

Ethical approval was obtained from the institutional ethical committee of Mahatma Gandhi Mission's (MGM) Medical College and Hospital, Aurangabad (approval number of MGM-ECRHS/2020/55, December 21, 2020). Informed consent was obtained from the parents of the patients.

## Results

The results of our observational descriptive study investigating the etiology of GDD at a tertiary care hospital in India are presented. Findings on demographic characteristics, proportions of various etiological categories, and genetic abnormalities detected are emphasized. This study may contribute to the understanding of the causes of GDD in children and guide further research in this area.

The study included 52 patients with GDD, with a median age of 15.5 months (interquartile range [IQR] = 33.25) (Table 1). The majority of patients (69.2%) were male, and 42.3% had a history of third-degree consanguinity of marriage. Cesarean section was the mode of delivery in 38.5% of cases. The mean birth weight was 2,318 g (standard deviation [SD] = 591 g), and at presentation, the median weight was 7.7 kg (IQR = 4.6), with 50% of patients being undernourished. The mean length was 75.56 cm (SD = 18.91 cm), and half of the patients had microcephaly. Macrocephaly was observed in only three patients. The mean developmental quotient was 37.41% (SD = 18.36). Clinical examination revealed hepatosplenomegaly in eight patients, hepatomegaly in five, congenital heart disease in four, and undescended testes in two patients. Neurological examination showed tone abnormalities and weakness to be common abnormalities.

**Table 1:** Demographic characteristics of patients (N = 52). *n*, frequency; LSCS, lower section Cesarean section; DQ, developmental quotient; HC, head circumference

Characteristic	Value
Age (Months), Median (IQR)	15.5 (33.25)
Male, *n* (%)	36 (69.2)
LSCS, *n* (%)	20 (38.5)
Birth weight (g), Mean (SD)	2318 (591)
DQ (%), Mean (SD)	37.41 (18.36)
Weight (kg), Median (IQR)	7.7 (4.6)
Length (cm), Mean (SD)	75.56 (18.91)
HC (cm), Mean (SD)	43.78 (4.48)
Microcephaly, *n* (%)	26 (50)

GDD is a heterogeneous disorder with various etiological categories. In this study, we classified the etiological categories of 52 patients into four groups (Table 2): prenatal, perinatal, postnatal, and unknown causes. Prenatal causes accounted for the largest proportion of cases, with genetic factors (32.7%), chromosomal abnormalities (7.7%), congenital neurological malformations (7.7%), and congenital infection (1.9%) being the subcategories. Perinatal causes were observed in a significant proportion of cases, with hypoxic-ischemic encephalopathy (HIE, 26.9%) and intracranial hemorrhage (7.7%) being the leading subcategories. Postnatal causes (3.8%) were less common and included chronic bilirubin encephalopathy and post-meningitic sequelae. In the rest of the cases, the etiology could not be identified (11.6%). So the etiological yield in our study was approximately 89.4%.

**Table 2:** Etiological profile of global developmental delay. HIE, hypoxic-ischemic encephalopathy; ICH, intracranial hemorrhage

Etiological category		n	%
Prenatal	Genetic	17	32.7
	Chromosomal	4	7.7
	Cerebral dysgenesis	4	7.7
	Congenital infection	1	1.9
Perinatal	HIE	14	26.9
	ICH	4	7.7
Postnatal		2	3.8
Unknown		6	11.6
Total		52	100

Based on the genetic testing results, a range of single-gene defects was identified (Table 3) as potential etiological factors for GDD. The following genetic variants were detected in the tested individuals: *IDS -* a hemizygous variant in exon 9 (c.1403G>A) causing MPS-II, an X-linked recessive disorder, was seen in two patients. *HEXA -* a homozygous variant in exon 7 (c.788C>T) causing Tay-Sachs disease, an autosomal recessive disorder. *GABRA1*(+) - a heterozygous variant in exon 11 (c.1397C>T) associated with developmental and epileptic encephalopathy-19, an autosomal dominant disorder. *SMPD1 -* two heterozygous variants in exons 4 and 6 (c.1311G>A and c.1757G>C) causing Niemann-Pick disease type A, an autosomal recessive disorder with one variant classified as pathogenic and the other as uncertain significance. *MPV17 -* a homozygous variant in exon 4 (c.210>A) causing mitochondrial DNA depletion syndrome 6 (hepato-cerebral type), an autosomal recessive disorder. *GBA -* a homozygous variant in exon 10 (c.1504G>T) causing Gaucher’s disease, an autosomal recessive disorder. *GLB1 -* two heterozygous variants, one in exon 11 (c.1122T>G) and one in intron 1 (c.75+3_75+4 del) causing GM1 gangliosidosis, an autosomal recessive disorder with one variant classified as pathogenic and the other as uncertain significance. *GFAP -* a heterozygous variant in exon 4 (c.715C>T) causing Alexander’s disease, an autosomal dominant disorder. *STXBP1 -* a heterozygous variant in exon 3 (c.166delA) associated with epileptic encephalopathy early infantile, an autosomal dominant disorder. *TSPOAP1*(-) - a homozygous variant in exon 22 (c.3994_4005delins) causing juvenile-onset progressive dystonia, an autosomal recessive disorder with uncertain significance.

**Table 3:** Single-gene defects identified in the patients. MPS II, mucopolysaccharidosis type II

Gene	Location	Variant	Zygosity	Disease	Inheritance	Classification
IDS	Exon 9	c.1403G>A	Hemizygous	MPS II	X-linked	Pathogenic
HEXA	Exon 7	c.788C>T	Homozygous	Tay-Sachs disease	Autosomal recessive	Uncertain significance
GABR A1(+)	Exon 11	c.1397C>T	Heterozygous	Developmental and epileptic encephalopathy-19	Autosomal dominant	Uncertain significance
SMPD1	Exon 4	c.1311G>A	Heterozygous	Niemann-Pick disease type A	Autosomal recessive	Pathogenic
SMPD1	Exon 6	c.1757G>C	Heterozygous	Niemann-Pick disease type A	Autosomal recessive	Uncertain significance
MPV17	Exon 4	c.210>A	Homozygous	Mitochondrial DNA depletion syndrome 6 (hepato-cerebral type)	Autosomal recessive	Pathogenic
IDS	Exon 9	c.1403G>A	Hemizygous	MPS-II	X-linked recessive	Pathogenic
GBA	Exon 10	c.1504G>T	Homozygous	Gaucher’s disease	Autosomal recessive	Pathogenic
GLB 1	Exon 11	c.1122T>G	Heterozygous	GM1-gangliosidosis	Autosomal recessive	Pathogenic
GLB 1	Intron 1	c.75+3_75+4 del	Heterozygous	GM1-gangliosidosis	Autosomal recessive	Uncertain significance
GFAP	Exon 4	c.715C>T	Heterozygous	Alexander’s disease	Autosomal dominant	Pathogenic
STXBP 1	Exon 3	c.166delA	Heterozygous	Epileptic encephalopathy early infantile	Autosomal dominant	Pathogenic
TSPOA P1(-)	Exon 22	c.3994_4005delins	Homozygous	Dystonia	Autosomal recessive	Uncertain significance

**Figure 1:**
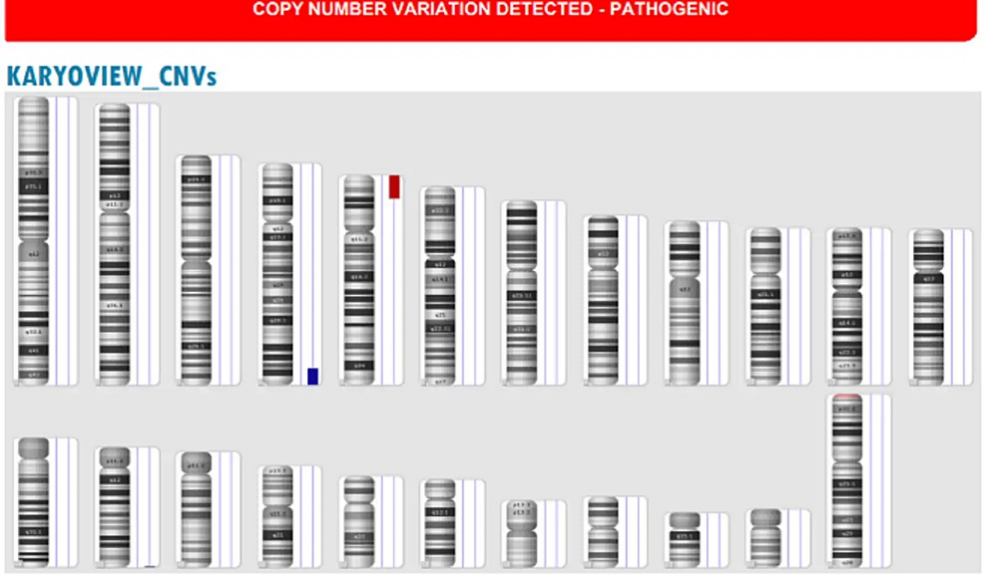
Karyotype of a patient with GDD. The cytogenomic microarray analysis showed a loss (19.97 Mb) involving chromosome 5 at cytoregion p15.33 to p14.3, and also a gain (14.06 Mb) involving chromosome 4 at cytoregion q34.2 to q35.2. GDD, global developmental delay

We conducted chromosomal tests on four children with dysmorphic features. Three of the children were diagnosed with Down's syndrome: one with trisomy 21 (47, XX+21), one with Robertsonian translocation (46, XY, Der (14; 21) (Q10; Q10) +21), and one with mosaicism (47, XX, +21(18)/46, XX (2)). The remaining patient had a microdeletion and a gain, which were detected through microarray testing. Specifically, the results showed arr[GRCh37] 4q34.2q35.2(176893733_190957460)x3, 5p15.33p14.3(113577_20088128)x1 (Figure 1).

**Figure 2:**
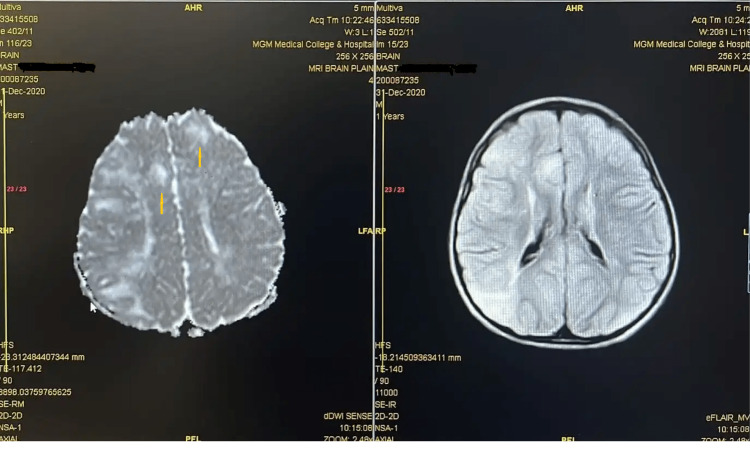
Cortical tubers in a patient with tuberous sclerosis.

Enzyme assay confirmed *N*-acetylhexosaminidase A enzyme deficiency in one patient diagnosed with infantile Tay-Sachs disease and a sphingomyelinase deficiency in another patient diagnosed with Niemann-Pick disease. The diagnosis of tuberous sclerosis was established in three patients based on clinical features, neurocutaneous markers, and MRI brain findings consistent with the presence of cortical tubers (Figure 2).

**Figure 3:**
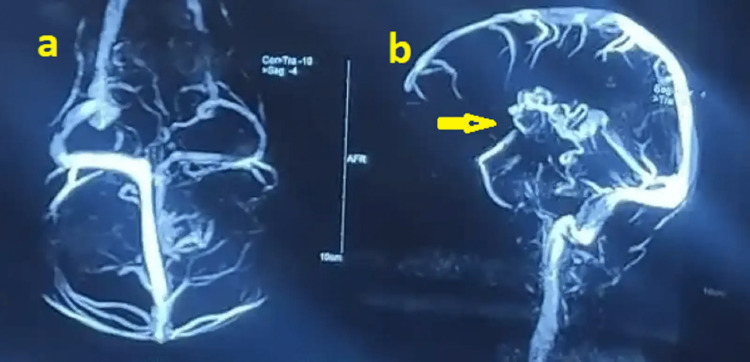
Magnetic resonance venography showing arteriovenous malformation: (a) venous drainage to the left internal cerebral vein, the vein of Galen, and the straight sinus; (b) multiple dilated vascular channels forming a tuft in the left caudate nucleus, ganglion-capsular region, and thalamus.

Several nongenetic prenatal causes were identified in the study. Among them, four children had congenital malformations detected on MRI brain scans, including one case of arteriovenous malformation (Figure 3), one case of Dandy-Walker syndrome, and two cases of corpus callosum agenesis. One child was diagnosed with congenital toxoplasmosis based on a consistent clinical history, physical examination, and MRI findings.

**Figure 4:**
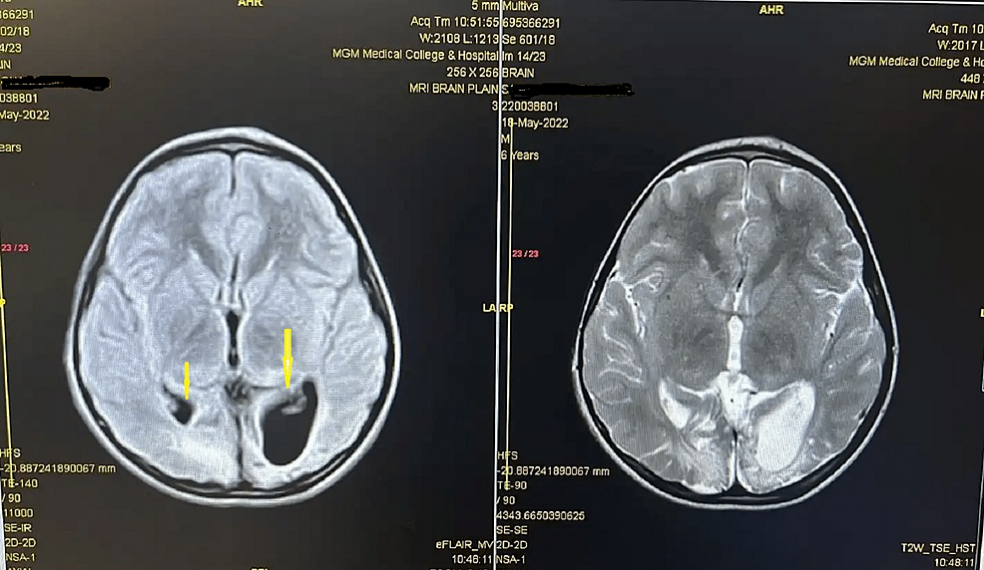
Neurological changes in a patient with HIE. HIE, hypoxic-ischemic encephalopathy

The most common perinatal cause (26.9%) was HIE, and four children had developmental delay secondary to intracranial hemorrhage which was diagnosed based on the patients' perinatal history, need for resuscitation, neonatal ICU (NICU) admission, presence of seizures, neurological abnormalities on examination, and consistent MRI brain findings (Figure 4).

Two children were classified as having postnatal causes of developmental delay: one had post-meningitic sequelae, while the other had sequelae of chronic bilirubin encephalopathy. Both diagnoses were made based on their clinical history, physical examination, laboratory test records, and MRI brain findings. Six children remained classified as having an unknown etiology for their developmental delay, as the specific disease causing their condition could not be ascertained. In these cases, MRI brain findings were nonspecific, and genetic tests were inconclusive.

## Discussion

In this observational study conducted at our tertiary care hospital, our objective was to determine the etiological profile of GDD. A total of 52 patients underwent a comprehensive workup, and we were able to identify the etiology in 89% of the cases. Among the identified causes, prenatal factors were the most prevalent, accounting for 50% of the cases, followed by perinatal factors at 34.6%. Conversely, postnatal causes were relatively rare, observed in only 3.8% of the cases. It is noteworthy that despite thorough investigations, the etiology remained unidentified in 11.6% of the patients. These findings shed light on the varying etiological patterns contributing to GDD, with prenatal and perinatal factors playing significant roles, while highlighting the challenges in identifying the underlying causes in a subset of patients.

We observed that genetic causes were identified in 17 (32.7%) of the cases, while chromosomal abnormalities were found in four (7.7%) patients. Consistently, genetic factors have been recognized as significant contributors to global developmental delay across multiple studies. For instance, Han et al. reported genetic causes in approximately 60% of cases of GDD/intellectual disability [5], while Chen et al. and Gowda et al. identified genetic factors in around 19% [6] and 41% [7] of cases, respectively. A study by Bandara and Munasinghe revealed genetic/chromosomal causes in 3.8%, possible genetic causes in 5.9%, and possible metabolic causes in 2.2% of cases [8]. Tikaria et al. reported chromosomal abnormalities, including Down's syndrome, in approximately 15% of cases [9], while Ozmen et al. found chromosomal anomalies in about 9% of cases [10]. Recent evidence recommends genetic testing as a first-line investigation for children with unexplained GDD, especially if an exogenous cause is not already established [11]. These findings emphasize the importance of cytogenetic evaluations in diagnosing and understanding developmental delay. It is worth noting that variations in the proportion of genetic causes could be attributed to factors such as sample size, access to genetic testing, available resources, and geographic location.

In our study, we identified intrauterine cytomegalovirus (CMV) infection in one (1.9%) patient and cerebral malformations in four (7.7%) patients as significant prenatal causes of developmental delay. Consistent with previous research, intrauterine infections have been recognized as contributing factors to developmental delay. The study by Ozmen et al. reported that approximately 2% of cases were attributed to intrauterine infections [10], while the study by Tikaria et al. found the proportion to be 4% [9]. These findings underscore the importance of considering infections during pregnancy as potential contributors to developmental delay. The research by Tikaria et al. reported cerebral dysgenesis in approximately 11% of cases [9], while the study by Ozmen et al found that congenital anomalies accounted for around 18% of cases [10]. These studies highlight the significance of comprehensive prenatal screenings and evaluations to detect congenital malformations and their association with developmental delay.

In our study, perinatal causes emerged as the next important etiological category, with HIE identified in 26.9% of cases and intracranial bleeding in 7.7% of cases. These findings align with the existing literature, which consistently highlights the contribution of perinatal factors to GDD. The reported proportions vary across studies, ranging from 20.4% [2] to 63% [12], further emphasizing the significance of these factors. Our results are in line with other studies reporting notable proportions, such as 24% [7] and 31.1% [13]. Within the perinatal causes, HIE stood out as the leading factor underlying GDD, with prevalence ranging from 10% [14] to 44.5% [15] across different studies. These findings are consistent with previous research, which has also reported substantial proportions, including 14% [9], 21% [10], 27.6% [8], and 41% [16]. These findings underscore the importance of perinatal factors and the need for appropriate management during delivery to minimize the risk of developmental delay.

Postnatal causes were relatively rare in our study, with one patient exhibiting post-meningitic sequelae and another with bilirubin encephalopathy. These findings align with previous studies reporting postnatal causes as less common contributors to GDD, with proportions ranging from 2.4% [17] to 9% [13]. Notably, Nguefack et al. reported that meningitis accounted for approximately 6.5% of cases [16] with post-meningitic sequelae, while 6.5% of cases were attributed to sequelae of kernicterus. Consistent with these observations, Bandara and Jha found post-meningitic sequelae in 7% [8] and 10% [15] of their respective study populations. These findings highlight the importance of early diagnosis and appropriate treatment to prevent long-term neurodevelopmental complications.

Our study achieved an etiological identification rate of 88.4%, aligning with the reported yields in comparable studies. However, the reviewed studies exhibited substantial variability in etiological yield, ranging from 58.4% [8] to 92.3% [12], highlighting the impact of different research settings on diagnostic success. A retrospective chart review by Majnemer and Shevell identified an etiological diagnosis in 63.3% of cases [14], while a study by Ozmen et al. in Turkey involving 247 children found a diagnosis rate of 64% [10]. Additionally, Jha, Tikaria et al., Koul et al., and Gowda et al. reported diagnostic yields of 71% [15], 73% [9], 71.8% [18], and 88% [7], respectively. Collectively, these studies emphasize the broad range of etiological yields and underscore the significance of comprehensive assessments to uncover and address the underlying causes of developmental delay. Ongoing exploration and understanding of these unidentified factors will provide invaluable insights for refining diagnostic strategies and implementing targeted interventions in the future.

Our study has certain limitations that should be acknowledged. First, the small sample size may have restricted the generalizability of our findings. Second, the single-centered nature of our study might have introduced site-specific biases and limited the range of identified etiologies. Conducting a larger, multicentric study involving a more diverse population would offer a broader understanding of the etiologic profile of global developmental delay in children. Moreover, the use of screening for developmental assessment in our study may have influenced the results’ accuracy and precision.

We humbly propose some future directions. First, there is a need to establish a comprehensive and accessible database of etiological profiles for global developmental delay, especially in developing and resource-poor countries. Such a database would facilitate early diagnosis, improve treatment planning, and enhance outcomes for children with developmental delays worldwide. Secondly, efforts should be made to develop a uniform etiological classification system that enables consistent comparisons across studies, as also proposed by Jimenez-Gomez and Standridge [19]. This would facilitate meta-analyses and inform evidence-based policies and interventions.

## Conclusions

Prenatal causes, including genetic and chromosomal abnormalities, are common in GDD. The utilization of genetic testing enhances etiological yield. HIE remains a significant factor and highlights the importance of perinatal care in preventing developmental delays. Large multicentric studies are needed for a comprehensive database of etiological profiles, and standardized categorization of GDD causes may help in effective comparisons across regions, studies, and healthcare strategies.

## Appendices

For the development of this paper, we prepared our manuscript. Then used ChatGPT for improving academic phrasing by simple prompts such as "Please suggest improvement in academic phrasing of the following sentence/ paragraph: “….”. The response of ChatGPT was reviewed and edited if required to suit the journal's requirements.

However, it's important to note that ChatGPT has its limitations. It may occasionally generate incorrect or misleading responses. It's also important to use critical thinking and verify the information provided by ChatGPT through reliable sources. It's essential to use it responsibly and complement its output with human judgment and expertise.
